# A diaphragmatic hernia in a traumatic patient simulating a hemorrhage: A case report

**DOI:** 10.1016/j.tcr.2023.100754

**Published:** 2023-01-04

**Authors:** Khulud Aolayan, Turki Almohammadi, Abdulrahman Alotaibi

**Affiliations:** a Department of Surgery, King Fahad Hospital, Medina, Saudi Arabia; b Department of Surgery, Faculty of Medicine, University of Jeddah, Jeddah, Saudi Arabia

**Keywords:** Traumatic diaphragmatic rupture TDR, Trauma, Hemothorax, Road traffic accident, Diaphragmatic hernia

## Abstract

A traumatic diaphragmatic rupture occurs in approximately 5 % of all trauma cases, making diagnosis difficult. Images can be used for most diagnoses; however, some can be detected intraoperatively. Based on its presentation, mechanism, side, diagnostic modality, and surgical approach, the diaphragmatic hernia can be discussed from several perspectives. In this report, we present the case of a 39-year-old female who suffered a rupture of her right diaphragm following a motor vehicle accident. Her symptoms mimic those of hemorrhage. A repair was performed through an abdominal approach, and the patient was discharged from the hospital without complications.

## Introduction

There is a prevalence of 0.8–5.8 % of traumatic rupture of the diaphragm with blunt trauma, most of which is caused by high-speed motor vehicle accidents and falls from heights [1]. Nearly 69 % of traumatic diaphragmatic ruptures (TDR) occur on the left side, and the stomach is the most frequently herniated organ, while 24 % occur on the right side and 15 % occur bilaterally [2].

In 1951, the first report of a rupture of the left diaphragm was published [3]. Generally, TDR presents two different types of symptoms, acute with immediate symptoms or chronic with delayed symptoms [1]. In the TDR, there can be a variety of symptoms that can compromise the diagnosis, such as tachypnea, shortness of breath, chest pain, and decreased air entry in the affected side, which can mimic hemothorax or pneumothorax [4]. Recently, a few case studies and reports have attempted to collect consensus on TDR and best management practices. We present a case of a 39-year-old female with TDR following a motor vehicle accident MVA.

## Case report

**Fig. 1:**
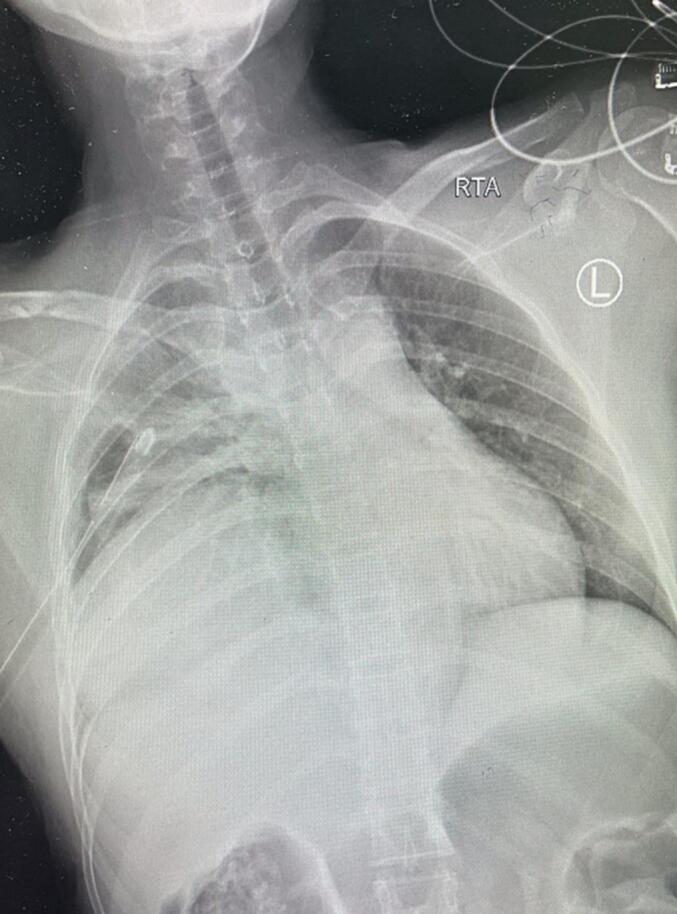
A chest x-ray indicates opacities on the right side of the chest with mid-clavicular fracture and intercostal tube in place.

We received a 39-year-old female admitted to our Emergency Department with a GCS of 15/15 and borderline vital signs. The patient was tachycardic and tachypneic, complaining mainly of the chest and right shoulder pain. Abdominal examination was unremarkable as well as the secondary survey. The chest x-ray showed a fractured right clavicle and hemothorax (Fig. 1).

**Fig. 2:**
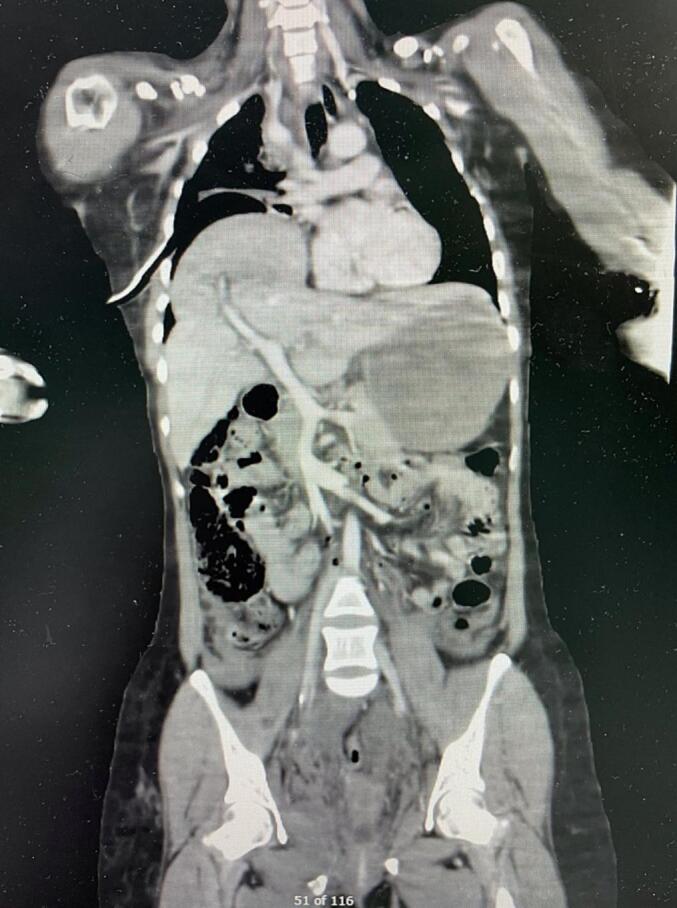
A CT scan with a coronal view demonstrates the right dome of the liver inside the chest with mediastinum shift.

**Fig. 3:**
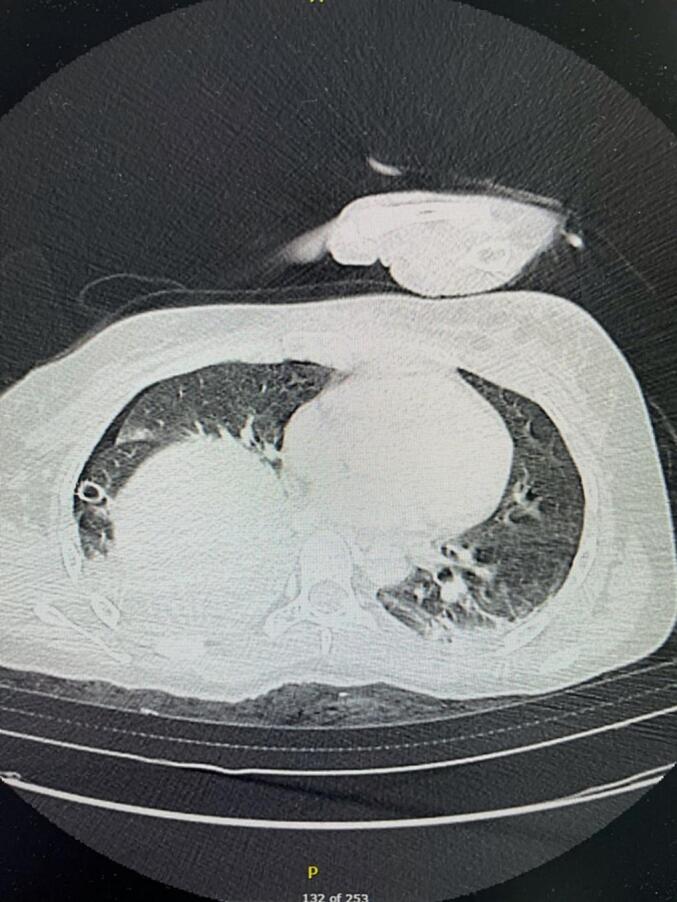
A CT scan in the transverse plane displays the right dome of the liver inside the chest cavity.

A right intercostal tube was immediately inserted, and approximately 700 ml of blood were drained. While the patient blood pressure remained borderline following fluid and blood resuscitation, a CT scan with contrast was performed. CT scan revealed diaphragmatic rupture and liver herniation, resulting in right lung volume loss and mediastinal shift (Fig. 2, Fig. 3).

Exploratory laparotomy was performed, and the herniated section of the liver was reduced. There was no severe injury or laceration to the lungs seen upon inspection of the right thoracic cavity. Polypropylene sutures were used to repair the diaphragmatic tears in two layers. Acute pancreatitis caused by the trauma complicated the patient's postoperative course, which was treated conservatively until the lipase level returned to normal. The patient was discharged home on day 19 postoperative.

## Discussion

An acute diaphragmatic rupture, particularly on the right side, is rare. It can be congenital or traumatic, with only 5 % of trauma victims experiencing this condition [5]. The right side of the diaphragm is less likely to suffer a rupture because protected by the liver [6]. The provisional diagnosis in the present case was right hemothorax, particularly with clavicular fracture and blood drained after the insertion of a chest tube. A chest x-ray is not the modality of choice to diagnose TDR as it can detect only 37 % of left-side diaphragmatic rupture [7]. Regarding diagnostic value, CT abdomen with IV contrast is the most sensitive and accurate test; it identifies the site, size, and complications related to TDR [1], [5]. In 15 % of all cases, the diagnosis of TDR can be missed. A case was reported in which the diagnosis was made after two years had passed since the trauma occurred [8]. A second case was diagnosed after 6.5 years of trauma [9]. Although there was no evidence of pancreatic herniation in the present case, some have reported acute pancreatitis following the herniation of the head of the pancreas because of penetrating trauma [10].

.While there is no consensus on the best surgical approach, some believe that the abdominal approach is more beneficial than the thoracic approach [7]. The minimally invasive approach suggests by some authors if the patient is stable, and TDR is questionable [11].

Table 1 shows some cases reported in the literature.

**Table 1:** A review of some reported cases of blunt diaphragmatic rupture.

Years	Cases no.	mechanism	site	Diagnosis	Access/approach	Morbidity/mortality
1998	1	Blunt	Right	CT scan	Open/abdominal	Pleural effusion/none [12]
2009	1	Blunt	Right	Intraop.	Open/abdominal	None [1]
2013	21	17 blunt	19 Left	CT scan	Open/abdominal	None [2]
2017	6	1 blunt	Left	CT/X-ray	Open/Both	None [5]
2017	1	Blunt	Left	CT scan	Open/abdominal	None [13]
2020	1	Penetrating	Left	CT scan	Open/abdominal	None [10]
2020	1	Blunt	Left	CT scan	Open/abdominal	Pleural effusion/ none [4]
2020	1	Blunt	Left	CT scan	Lap / abdominal	None [9]
2021	1	Blunt	Right	X-ray	Open/thoracic	None [11]
2022	1	Blunt	Left	CT scan	Open/abdominal	None [8]

## Conclusion

Right-side Traumatic Diaphragmatic rupture is a rare condition that carries low morbidity and mortality rate upon early recognition. Diaphragmatic rupture should be considered by the surgeon when addressing a right-side hemothorax. An abdominal approach is usually the most appropriate, particularly in polytrauma cases.

## CRediT authorship contribution statement

Khulud Aolayan, Turki Almohammadi, and Abdulrahman Alotaibi conceptualized the case report and collected the data, wrote the entire manuscript, and approved the final version of the manuscript.

## Declaration of competing interest

The authors declare no competing interests.
